# Editorials

**Published:** 1909-03

**Authors:** 


### EDITORIALS

The Business Office of the JOURNAL-RECORD is 1 1-2 to 5 1-2 South Broad Street.
The Editorial Office is 1014-15 Century Building.
Address all Business Communications to *Journal-Record of Medicine*, 1 1-2 to 5 1-2 South Broad Street.
Make remittances payable to *THE JOURNAL-RECORD OF MEDICINE.*
On matters pertaining to the Editorial and Original Communications, address Edgar G. Ballenger, M. D., Atlanta, Ga.
Reprints of Original Articles will be furnished at cost price. Requests for the same should always be made in THE MANUSCRIPT.
We will present, postpaid, on request, to each contributor of an original article, twenty (20) marked copies of THE JOURNAL-RECORD OF MEDICINE containing such article.

### THE PERSONAL EQUATION.

"The proper study of mankind is man."—Pope.

Just now, while the followers of Paul Dubois, Ellwood Worcester and Mrs. Eddy are indulging in polemics, that make both the sparks and the fur fly, the thoughtful physician may glean from this wilderness of words a valuable harvest.

Our many and excellent medical colleges can equip the candidates for the practice of medicine with theories galore; can hold out to him the lamp of knowledge, glowing from the accumulated stores of centuries of the midnight oil; but, unless he studies men, unless he hears some of those "Sermons in stones," or reads some of those "Books in running brooks;" unless, in his dealings with the sick he exercises that homely trait, called by the late Joe Brown "judgment," he can never hope to be eminent in his profession.

The common paraphrase from Saint Paul's words, "Milk for babies and meat for strong men," may well be amplified in our everyday life.

The laity are reading now as never before. Studies in the various phases of psychological research are being furnished by enterprising periodicals in a form attractive and easily understood, while the various cults, each claiming to have discovered a new and basic principle, find no difficulty in reaching the ear of the public.

Not so very many years ago, when a doctor was consulted, his remedies were taken without question, and his *ipse dixit* was sufficent reason for the procedure set in motion. The whys and wherefores did not interest the patient, so long as relief was obtained.

Many of the most charming pictures drawn by novelists have portrayed the family physician, secure in the confidence, respect and love of his clientele; whose statements admitted no argument, whose decisions were appealed to no higher court.

Some of those old worthies possessed not a tithe of the scientific attainments held by our present-day medical lights, but they, in their spheres, wielded an influence almost autocratic in its power.

Then that other strong type, the general practitioner, (who it is freely predicted will soon be as extinct as the dodo) with his intimate acquaintanceship in his community, marks the antithesis of that ultra sect, who regards sick people as so many human units, to be judged by their qualities of tissue-resistance.

Far be it from me to decry any of the modern means of diagnosis or treatment. We surely need them all. Nor would I minimize the inestimable value of the labors of Virchow, Pasteur, Metchnikoff, Wright and others, that host of unselfish investigators, to whom the world owes so much.

What I plead for is a greater consideration for the personal equation, and with it the *temperament,* that climate of the mind, as it is called by Weir Mitchell.

When we can tap the hidden springs in our patients' emotions; locate the jarring elements of personality, probably making up a part of some vicious circle; when we can make the sufferers feel that they are getting not only adequate medical treatment, but in addition a sincere study of their individualities, our influence for good will be greatly strengthened, and the gratifying results will abundantly justify our efforts.

In considering idiosyncrasies of body, let us also seek for those of thought. In bolstering up weaknesses of functions, let us not neglect weaknesses of will power. In stimulating the flagging energies of vital organs, let us not forget that discouragement or disappointed ambition may be a causative factor of importance. Most of all let us, as far as consistent with the truth, ring a note of hope and good cheer with every prescription, with every word of advice.

Thus, in deeper study of the human side of those who come to us in their infirmities, whether real or imaginary, we can more fully measure up to what is required of the medical profession, command greater confidence from the public at large, and render unprofitable the calling of these healers, who promise so much, and who fail entirely to realize that "A little learning is a dangerous thing."

G. M. N.

---

### SOME SUGGESTIONS REGARDING APPLICATION OF THE ROSE BELT.

In the February issue of the Journal-Record the writer indulged in some remarks concerning gastroptosis in tubercular patients, but did not attempt to give any treatment for this condition.

The general management of gastroptosis, with the frequent accompanying ptoses of the other abdominal viscera, is a broad subject—too broad to be adequately described here.

This downward mal-position, being to a great extent caused by atony, the principal and rational means of correcting it would be mechanically supportive, and many forms of support have been advocated and used with more or less success.

Several years ago Dr. Achilles Rose, of New York, began using a special form of adhesive plaster made on moleskin, and applied with an upward pull to the lower abdomen, his ideas and methods being very fully brought out in an inexpensive little monograph entitled "Atonia Gastrica," by Rose and Kemp, and published by Funk & Wagnalls Co., 1905.

The technic of applying this belt is fairly well described also in Conheim's late book on the digestive canal. There are, however, some modifications devised later by Drs. Robert Kemp and Theodorus Bailey, of New York, which give greatly improved results.

Both Conheim and Dr. Rose himself, in an article appearing in the Medical Fortnightly, of March 25th, portray the belt being applied while the patient is standing erect. Dr. Kemp, in putting it on, requires the patient to lit down, while Dr. Bailey goes further, elevating the hips six or more inches, thereby facilitating the gravitation of the ptosed organs into the upper part of the abdominal cavity. This permits the belt to be fitted on more snugly and effectively.

Any physician, who has been accustomed to placing this belt in position with the patient standing, will find that, while it entails a little more time and care to get it smoothly fitted with the patient recumbent and the hips elevated, the satisfactory results obtained will easily justify the increased efforts.

The writer wishes, therefore, to commend to the profession the desirability of correcting by the use of this belt, whenever possible, the various atonic ptoses of the abdominal viscera, for often it will be found that, with these in proper place, a long train of perplexing symptoms will either disappear, or be markedly improved.

G. M. N.

---

### RESOLUTIONS ON THE DEATH OF DR. C. C. STOCKARD.

---

One by one our co-workers answer the Final Roll Call and lay down their armor in peace to enter the Life beyond. Dr. Charles Cecil Stockard, our friend and professional brother, answered that summons in the full fruition of his professional life and usefulness.

To know him was to love and respect him.

He was a true Christian physician, believing not only in the spiritual life beyond, but also in living that life here, proving true to his church, his family, his professional brethren and to suffering humanity.

He did much to establish an ethical, legitimate, professional basis of the treatment of the drug habitue and inebriate.

Dr. Stockard was one of the pioneers in the establishment of a private sanitarium or infirmary in the city of Atlanta, owning and operating one at the time of his death. As a small token of our regard and esteem, be it

Resolved, That in the death of Dr. C. C. Stockard, the Fulton County Medical Society has suffered a material loss, the profession and humanity a tried and true friend. That we extend to his widow and family our sincere sympathy and sorrow.

That a copy of this report be spread upon the minutes, a copy be sent to Mrs. Stockard and that it be published in the daily papers.

Respectfully submitted,

R. R. KIME,
W. L. CHAMPION,
L. C. FISCHER.

---

### RESOLUTIONS OF SYMPATHY FROM THE FULTON COUNTY MEDICAL SOCIETY TO THE WIFE AND LOVED ONES OF DOCTOR WILLIAM BUCKINGHAM ARMSTRONG.

Since the Wise and Omnipotent Father has seen fit to remove from among us Doctor William B. Armstrong, be it resolved by the members of this society that we have sustained on irreparable loss in the death of our beloved friend, whom we had learned to so love and esteem, and that we extend to his wife and family our deepest and most heartfelt sympathy.

Be it further resolved, That we know words are both lacking and inadequate for the expression of our feelings in so sad an hour, and that the loss of our friend, a noble gentleman, a skillful surgeon and one who was ever eager to lend a helping hand to the afflicted and suffering, can only be compensated for in the solace that He who was "a Man of Sorrow and acquainted with grief" directs our lives for the best and good of all.

Be it further resolved, That copies of these resolutions be sent to the wife and the mother of Dr. Armstrong, be spread on the minutes of this society and be published in the daily papers.

C. R. ANDREWS,
A. W. STIRLING,
J. H. JOHNS.

---

### RESOLUTIONS ON THE DEATH OF DR. W. B. ARMSTRONG, BOARD OF THE GRADY HOSPITAL.

Whereas, God in His wisdom has seen fit to remove from our Board our beloved friend and professional brother, Dr. W. B. Armstrong, and

Whereas, In his death we have lost a friend and colleague who by his noble life has not only endeared himself to us but to the profession and the people whom he served, and

Whereas, This Hospital has lost an able surgeon and true friend:

Whereas, That a page in our Minutes be set aside to the memory of this modest, unassuming gentleman, whose loss has caused us inexpressible grief.

Resolved, That our deepest sympathy be tendered to the various members of his family in this time of their affliction.

Signed,

W. A. CROW,
W. S. GOLDSMITH,
C. W. STRICKLER.

